# An estimate of the impact rate on Mars from statistics of very-high-frequency marsquakes

**DOI:** 10.1038/s41550-024-02301-z

**Published:** 2024-06-28

**Authors:** Géraldine Zenhäusern, Natalia Wójcicka, Simon C. Stähler, Gareth S. Collins, Ingrid J. Daubar, Martin Knapmeyer, Savas Ceylan, John F. Clinton, Domenico Giardini

**Affiliations:** 1 Institute of Geophysics, ETH Zurich, Zurich, Switzerland; 2 Department of Earth Science and Engineering, Imperial College, London, UK; 3 Earth, Environmental, and Planetary Sciences, Brown University, Providence, RI USA; 4 DLR Institute of Planetary Research, Berlin, Germany; 5 Swiss Seismological Service, ETH Zurich, Zurich, Switzerland

**Keywords:** Seismology, Inner planets, Seismology

## Abstract

The number density of impact craters on a planetary surface is used to determine its age, which requires a model for the production rate of craters of different sizes. On Mars, however, estimates of the production rate of small craters (<60 m) from orbital imagery and from extrapolation of lunar impact data do not match. Here we provide a new independent estimate of the impact rate by analysing the seismic events recorded by the seismometer onboard NASA’s InSight lander. Some previously confirmed seismically detected impacts are part of a larger class of marsquakes (very high frequency, VF). Although a non-impact origin cannot be definitively excluded for each VF event, we show that the VF class as a whole is plausibly caused by meteorite impacts. We use an empirical scaling relationship to convert between seismic moment and crater diameter. Applying area and time corrections to derive a global impact rate, we find that 280–360 craters >8 m diameter are formed globally per year, consistent with previously published chronology model rates and above the rates derived from freshly imaged craters. Our work shows that seismology is an effective tool for determining meteoroid impact rates and complements other methods such as orbital imaging.

## Abstract (web summary)

A subset of seismic events recorded by InSight’s seismometer attributed to impacts was used to derive a new estimate of the current impact rate on Mars. The results suggest a rate higher than predicted by orbital imaging, but consistent with theoretical rates.

## Main

The current meteoroid impact rate on Mars is vital for determining accurate absolute ages of surfaces throughout the Solar System^1–3^. The rate of impacts is also a function of the size distribution of small asteroids with implications for the formation of the Solar System as well as for the hazard to spacecraft. The current Martian cratering rate of craters <60 m has been estimated from repeated satellite imaging (for example, refs. ^4,5^), but such observations are limited by camera resolution and dust coverage. New impact sites are usually first identified by darkened surrounding areas, many times larger than the craters themselves, where dust has been disturbed during impact. This requires the target area to be dust-covered to some extent. Extrapolation of the crater density in dusty areas to the full planet requires a global model of dust mobility, for which the thermal inertia is a proxy^6,7^. As most areal coverage was obtained with the medium-resolution context camera onboard the Mars Reconnaissance Orbiter spacecraft^8^, current imaging-based cratering rates are most uncertain for smaller craters <8 m in diameter. An alternative approach is based on lunar crater chronology models. These are determined from the crater density of large (kilometre-scale) craters and are calibrated with radiometric lunar sample ages for geological timescales (for example, refs. ^1–3,9^). The extrapolation to Mars and to small, frequently forming, crater sizes requires accounting for the increased meteor flux on Mars compared to the Moon due to its proximity to the asteroid belt as well as the filtering effect of atmospheric deceleration of the smallest impactors. For craters <60 m, the contemporary cratering rates estimated from chronology models are 2–3 times higher than the imaging-based rate^5^.

**Extended Data Fig. 1:**
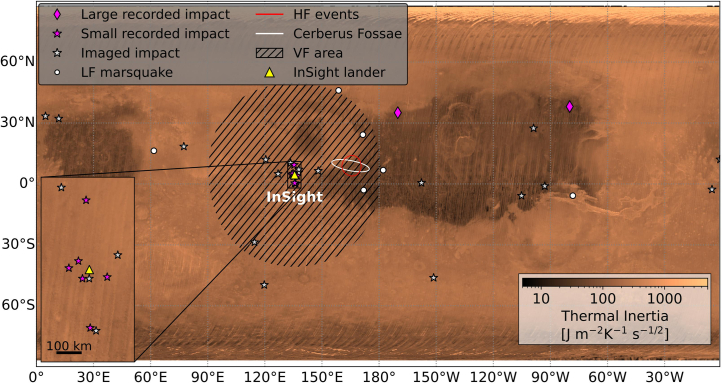
Map of Mars with impact and marsquake locations. Shown are the InSight location (yellow triangle), low frequency marsquake locations (white dots), and small and big impacts associated with seismic events since the start of the InSight mission (pink stars and diamonds, respectively). The white ellipse marks the Cerberus Fossae region, where many low frequency quakes originate. The red circle marks the HF event location region^17^. The hatched area shows the VF event source region (45^∘^, ≈ 2600km from InSight). Grey stars mark imaged impact craters not associated with a seismic event. The background shows thermal inertia data from the Mars Global Surveyor Thermal Emission Spectrometer^6^. A zoom inset shows the locations of impacts close to InSight in more detail.

The NASA InSight mission deployed a seismometer (SEIS)^10^ in Elysium Planitia. The instrument is capable of providing a third, independent estimate of the current impact rate, unconstrained by camera resolution and dust coverage. Meteoritic impacts create seismic waves like those of tectonic marsquakes and can be classified and counted^11^. From the first days of the mission, dedicated imaging of the area surrounding InSight detected new craters^12^, but only late in the mission were fresh craters unequivocally associated with seismic events^13,14^. By the end of the mission, eight seismic events recorded by SEIS had been identified as impacts by association with newly formed craters in orbital images^15^. Extended Data Fig. 1 shows a global map of Mars with seismically detected impact locations, as well as some known craters that formed while InSight was operational on Mars but have not so far been associated with seismic events. Identifying impacts from seismic data requires discrimination between tectonic events and impacts. The six seismically detected impacts closest to the InSight lander all belong to a type of marsquakes in the InSight catalogue known as very-high-frequency (VF) events. Moreover, these six are also among the closest VF events. Extended Data Table 1 gives all impact-related seismic events with event and crater properties, including two distant craters. To date, no seismic events have been associated with an impact crater of diameter between 12 and 140 m. No impacts located at intermediate distances from the lander (300–3,000 km) have been associated with a seismic event. This raises a natural question of whether more, and potentially all, VF events were generated by meteoroid impacts. Here we first consider whether the characteristics of the VF events are consistent with an impact origin. Assuming that all VF events are generated by impacts, we then use the population of VF events to derive the first seismically derived estimate of the current Mars cratering rate.

## Are all VF events impacts?

Why do we focus on VF events among all the seismic events that InSight has recorded? Seismic events are categorized by the Marsquake Service (MQS) into groups, notably VF, high frequency (HF) and broadband (BB), as described in Methods. HF and VF events both show a strong coda indicative of a very shallow source^16,17^. VF events are characterized by substantial energy at very high frequencies, 5–30 Hz (ref. ^18^). Some of the closest ones have a signal dubbed ‘chirp’ in their coda and are associated with impacts (Methods). Given that, we consider whether the characteristics of the entire subgroup are consistent with an impact origin.

### VF content and envelopes

**Fig. 1:**
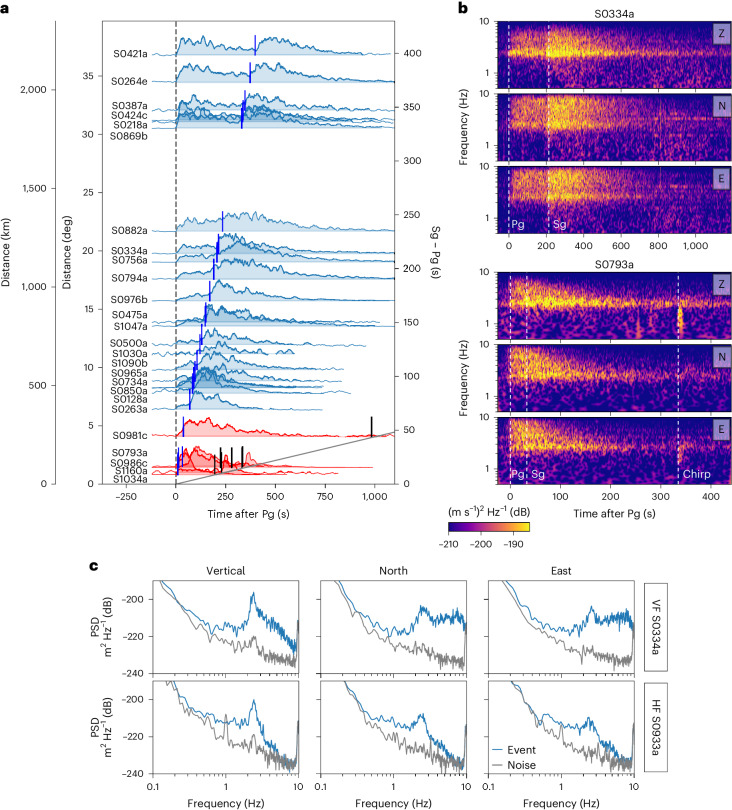
Overview of VF events. **a**, Envelopes of recorded VF quality B events sorted by distance, plotted from 120 s before to 1,100 s after the event. The envelopes are for the vertical component between 2.1 and 2.7 Hz, where, due to a local subsurface resonance at 2.4 Hz, the signal-to-noise ratio is typically highest^47,48^. They are normalized by their maximum amplitudes in that frequency band, aligned on the first (Pg) arrival. Marked are second (Sg) arrivals (blue line), chirp arrivals (where present; ‘Acoustic signal in VF coda’, black line) and the moveout of the speed of sound on Mars (240 m s^−1^, ref. ^41^, grey line). Confirmed impact events (red) are placed at the distance where the craters were found^15^. **b**, Velocity spectrograms for events S0334a (top) and S0793a (bottom) between 0.5 and 10 Hz. Phase arrivals are marked with vertical dashed lines. **c**, Displacement power spectral density (PSD) for a VF event (top, S0334a) and an HF event (bottom, S0933a). VF events have excess energy in the horizontal components (north (middle) and east (right)) at high frequencies compared with the vertical component. All three components of HF events have a similar spectral shape. The resonance peak at 2.4 Hz is visible most strongly on the vertical. All phase picks, plus event start and end, are taken from the MQS catalogue^21^.

**Extended Data Fig. 2:**
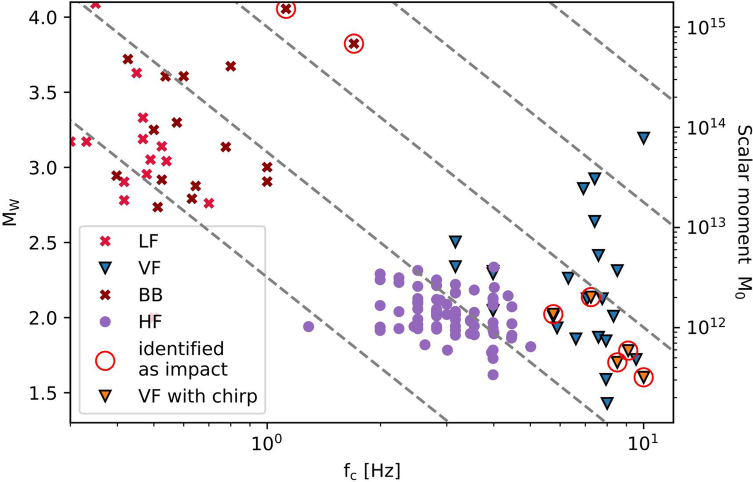
Distribution of corner frequency *f*_*c*_ over seismic moment M_0_ for the different types of marsquakes, following a fitting of P- and S-wave spectra with the model described in Stähler et al.^17^. LF (Low Frequency) and BB (Broadband) events are generally recognized to occur in Cerberus Fossae or other tectonically active regions within the deeper crust^16^. HF events have been described as occurring on shallow faults in Cerberus Fossae^17^, the circles mark marsquakes identified with fresh impact craters. The dashed lines mark the ${f}_{c}\approx {A{M}_{0}}^{-3}$ relation expected for tectonic sources^42^, with different values for *A*, depending on stress drop and local shear wave velocity. The distribution shows that from the spectral perspective, identified impacts stand out from the other marsquakes, but do not differ substantially from VF events.

The decisive difference between the VF and HF events is the former’s higher corner frequency. VF events are generally outside the scaling relation between magnitude and corner frequency found for other events^17^. That is, they have more HF energy than predicted for a marsquake of their size (Extended Data Fig. 2). The high corner frequency (>4 Hz) indicates a short source duration, consistent with a hypervelocity impact. The telltale signature of the VF events in the seismic record is a strong horizontal motion at frequencies above 5 Hz (ref. ^19^), which has been explained by a local resonance effect^20^ (for a spectral comparison of a HF and VF event, see Fig. 1c). HF events have corner frequencies below 2 Hz and, thus, not enough energy above 5–7 Hz to excite the horizontal resonance.

Over 3 yr of recording (February 2019 to June 2022), before power availability limited operations, InSight detected 70 VF events. Of these, 59 phase arrivals were identified and distances estimated (quality B or C) (catalogue v.12)^21^. The higher quality B VF event envelopes are shown in Fig. 1a, and spectrogram examples in Fig. 1b. Compared to the known impact events, the VF-event wave trains are longer in duration and have noticeably more distinct Sg arrivals, consistent with larger distances and, thus, travel times.

### Frequency–magnitude distribution

**Fig. 2:**
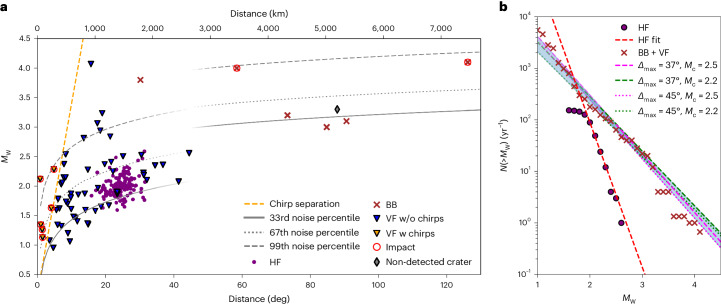
Magnitude–distance distribution and FMD of recorded VF and HF events. **a**, VF events with chirps (w chirps; yellow triangles) and without chirps (w/o chirps; blue triangles), as well as HF events (purple dots) and selected BB events (red X’s). Confirmed impact events^15^ are marked with red circles. A separation between chirp and non-chirp VF events is shown with a yellow dashed line. See the discussion in ‘Acoustic signal in VF coda’. Noise percentile levels (Extended Data Fig. 3 and ‘Background seismic noise’) are given by the solid (33rd percentile), dotted (67th percentile) and dashed (99th percentile) lines. As we assume that VF-like wave propagation trapped in the crust is possible only to 45° distance, the noise level at longer periods, relevant for the BB events, is used for larger distances. An imaged crater not detected by SEIS (grey diamond, 35 m crater constrained to 2 d; https://www.uahirise.org/ESP_076590_2180) was used to assess the noise levels. **b**, Cumulative number of events over all of Mars. The Gutenberg–Richter fits for ≥*M*_W_ were estimated as 2.0 (HF, purple dots and dashed red line), and 2.2 and 2.5 (VF and BB, red X’s and dashed and dotted lines, faded lines for *M*_W_ < magnitude of completeness). The blue-shaded area highlights the uncertainty between the different fits. VF and BB events are scaled to a global number from different cutoff distances, using a magnitude-dependent area correction, as explained in ‘Seismology event normalization’.

Figure 2a shows the magnitude–distance distribution of quality B and C VF events, HF events and selected BB events recorded by InSight. The BB events include the two large impact events (S1000a and S1094b), plus several other events (S0395a, S1102a, S1133c and S1153a) with similar characteristics (with more HF content ≥2.4 Hz than is typical for BB events) that are not associated with a known tectonic source region. Although not confirmed, they are potential candidates for impacts as well, given their unusual frequency content.

We also examined whether the spatial and temporal distribution of VF events is consistent with that of impacts. The former is expected to be randomly distributed over the surface of Mars. We found that the spatial distribution of VF-event sources is both consistent with an impact-generated source mechanism and clearly distinct from that of other event types (Methods). The temporal distribution is more difficult to assess. MQS detected all types of seismic events (low frequency/BB, HF and VF) at a 30–50% higher rate in the second year of the mission^22^ (Supplementary Information Section 1), for reasons that are, so far, not understood and are difficult to quantify statistically given the relatively short dataset from the single station. All seismically confirmed impacts occurred in the second year of the mission^13–15^. We conclude that the observed VF-event detection rate is not a strong argument against a stationary rate of impacts.

In addition to the spatial distribution, we examined whether the distribution of event sizes is consistent with that expected for impacts. The Gutenberg–Richter law (for example, ref. ^23^) describes the distribution of moment magnitude *M*_W_ and number of events *N* (frequency–magnitude distribution, FMD) as

1 $$N({\ge} {M}_{{{{\rm{W}}}}})=1{0}^{{a}_{{{{\rm{s}}}}}-{b}_{{{{\rm{s}}}}}{M}_{{{{\rm{W}}}}}},$$

where *a*_s_ is the productivity factor. Here *b*_s_, simply known as ‘the *b*-value’ to seismologists, gives the slope on a log–log plot. Tectonic marsquakes have a *b*-value ~1 (ref. ^24^). Figure 2b shows the FMD of both HF and VF plus BB events. The events are scaled for both area and time (Methods) and are, therefore, plotted as the total number of events per year over all of Mars. Note that ‘year’ and ‘day’ refer to the Earth definitions, unless otherwise stated. The *b*_s_-value was estimated using the Aki–Utsu maximum likelihood approach (described in, for example, ref. ^25^). Evidently, the HF events have a much steeper distribution at *b*_s_ = 2.81 than the VF plus BB events, which have a mean of *b*_s_ = 1.09. This further highlights that HF and VF events form separate classes of events, despite both having shallow-source characteristics.

Numerical modelling studies have shown that the seismic moment *M*_0_ scales linearly with impact momentum^26,27^ and as a power law with crater diameter *M*_0_ = *cD*^*n*^, where *n* = 3.3 represents an average between strongly cohesive material and a cohesionless sand. This relationship can be used to convert *b*_s_ into an equivalent slope for a diameter–frequency distribution, as used for cratering rates (see the derivation in Supplementary Information Section 2). This gives an ‘impact’ slope of *b*_i_ = 2.40, which lies within the slopes determined from previous studies for craters between 8 and 30 m in diameter^28^. The VF (plus selected BB) event FMD is, therefore, compatible with an impact origin.

The seismic analysis shows that on average, 300 ± 100 VF events of magnitude *M*_W_ > 2 and 30 ± 10 of *M*_W_ > 3 occur per year on Mars. In summary, these events are different in terms of their size-corner frequency distribution to all other events, are not associated with known tectonic features and are consistent with a random spatial distribution over the planet. A number of them, specifically all the closest ones, can be attributed to known new craters^13,15^.

Associating one of the more distant, chirp-less VF events with an observed new crater would greatly strengthen the argument that all VF events are impacts. Alas, targeted imaging campaigns are difficult for more distant events, as the unconstrained back azimuth leads to a very large search area. A recent pre-impact image is also necessary to constrain the impact date, which may not be available. However, even without this further confirmation, the case is strong that the unique VF marsquake class is consistent with impacts. It is, therefore, worthwhile considering the implications of attributing all VF events to meteoroid impacts.

## A seismically constrained impact rate for Mars

Size–frequency distributions (SFDs) of craters spanning a large range in diameters (metres to several kilometres) are complex due to the underlying complexity of the SFD of the impactors, size-dependent changes in crater formation and secondary cratering. Over a small crater size range, an SFD is approximated well by a single power law, as considered in this study. A power law for the cumulative number of craters *N* with diameter ≥*D* can be expressed as (for example, ref. ^29^):

2 $$N({\ge}D)=k{D}^{-{b}_{{{{\rm{i}}}}}},$$

where *k* is a productivity factor and the *b*_i_-value is the slope on a log–log plot of *N* versus *D*, where this equation produces a straight line. The *b*_i_-value is an important characteristic when determining the impact crater production function. The impactor size distribution should not vary spatially and, thus, does not require a normalization to the total area, making it easier to determine from incomplete datasets than the overall production rate *k* (ref. ^28^).

In cumulative impact crater counts based on orbital imaging, the crater size distribution shows a roll-off at small sizes where this power law is no longer applicable. This roll-off has been explained as a combination of camera resolution and atmospheric deceleration, ablation and fragmentation, which affect the smallest impactors^30^, although the relative contribution of the two factors is not known^5,31^. Smaller craters are also easier to miss in orbital images. Dust coverage and variations in the local geology can introduce further biases to the crater distributions.

The derivation of an impact rate from seismic observations requires several steps: (1) converting the seismic moment *M*_0_ into an estimate of the diameter of the crater that would have produced a signal of that size at that distance and (2) an area and time correction analogous to the area time factor (ATF)^5^ used in imaging-based cratering rates. We present two approaches for calculating a seismically constrained impact rate. One uses a seismological approach (referred to as ‘Seismology’) and the other a converted-crater diameter counting approach (‘Cratering’). The Seismology approach uses the Gutenberg–Richter distribution (Fig. 2) after applying an ATF based on seismological considerations (Methods) and converts this distribution into an impact rate after converting *M*_0_ to diameter. The Cratering approach converts each seismic event into a crater diameter. The craters are then counted and binned as would-be imaged craters and scaled by an appropriate ATF. A workflow diagram is shown in Supplementary Fig. 1.

## Impact rate estimates

### Crater diameter versus seismic moment relationship

To convert the moment magnitude distribution of VF events into a crater diameter size distribution, we used several established impact scaling relationships. The seismic moment for a specific impact scenario is uncertain, as it depends on the exact target properties and subsurface structure of the impact site.

**Fig. 3:**
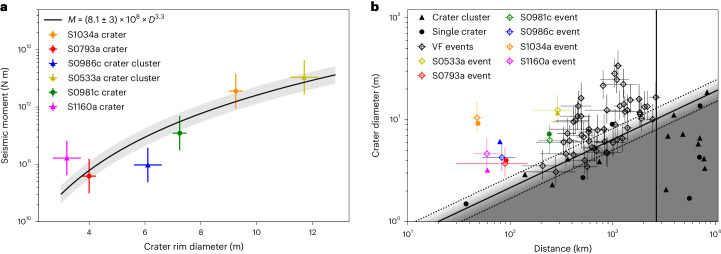
Overview of confirmed impacts and VF events detected by InSight. **a**, Seismic moment as a function of crater diameter. The coloured markers show the seismic moment measured at InSight and the observed crater sizes (circles for single craters and triangles for the effective diameter of crater clusters) for the six recently detected impacts listed in Extended Data Table 1 (refs. ^13,15^). The vertical error bars reflect the uncertainty in seismic moment magnitude derived using standard error propagation techniques. The horizontal error bars are given by the resolution of HiRISE images used to determine the crater sizes. The black line shows the least-squares fit to these events and the associated uncertainty (shaded area). **b**, Detectability of craters of a given diameter as a function of distance. The filled black circles and triangles are the observed, but not seismically detected, impact craters and crater clusters (respectively) that were constrained to have occurred during the InSight mission. The coloured filled-in markers indicate the observed and seismically detected craters and clusters, colour-coded by event name, as in **a**. The hollow diamonds in corresponding colours are the predicted crater sizes of detected impact events based on seismic moment magnitude. The black hollow diamonds show the distribution of predicted crater sizes for all other VF events. The solid black lines indicate the detectability curve. For distances up to 45° (~2,600 km, marked by the vertical black line), this is defined by the amplitude–distance scaling relationship. This curve defines the area in the ATF used to scale impact rates. Data are presented as mean values ± standard error derived using standard error propagation from uncertainties associated with a seismic moment versus crater diameter relationship and the spectral fit of the VF events.

As discussed in ‘Impact momentum scaling’, the seismic moment scales linearly with the impact momentum and as a power law with crater diameter *M*_0_ = *cD*^3.3^. We constrained the proportionality constant *c* to 8.1 × 10^8^ N m, using six small impacts seismically detected by InSight^13,15^ (Fig. 3a). Hence, we assume the following relationship between *M*_0_ and *D*:

3 $${M}_{0}=(8.1\pm 3.0)\times 1{0}^{8}{\left(\frac{D}{1\,{{\mbox{m}}}}\right)}^{3.3}{{\mbox{N}\,\text{m}}}.$$

Note that since this relationship is empirically constrained, any systematic error in the absolute value of *M*_0_ from the seismic analysis would be cancelled out. With this relationship, the VF-event FMD (equation (1)) and small-impact SFD (equation (2)) can be fully converted (Supplementary Information Section 2).

### Cratering approach

#### Predicted crater diameters

To predict crater sizes for all VF events, we used the scaling relationship between seismic moment and crater diameter (equation (3)). We considered only the VF events as they produced craters in the size range of the numerical simulations that underpin the seismic moment versus crater diameter relationship (1–30 m)^26,27^. Events that produce larger craters require more careful consideration of source depth, as described in ‘Cratering event selection’.

The resulting crater diameters for other VF events range between 3 and 40 m, which agrees well with the size range of craters on Mars that have formed in the last two decades^28^.

#### Impact detectability curve

The second element of this method is based on amplitude–distance scaling relationships for impacts, which shows that the peak P-wave amplitude scaled by impact momentum decreases with distance as *x*^−1.56^ (ref. ^13^). The vertical offset is derived in ‘Impact momentum scaling’. The resulting curve is shown in Fig. 3 and is used to calculate the area factor for nearby impacts, which is necessary when scaling impact rates. We assumed that VF events are detectable if they lie above this curve and at distances <45°, resulting in a total of 58 events. Figure 3b also shows craters and crater clusters with image-constrained formation dates during InSight’s operations that were not detected seismically. Most of these craters lie below the detectability line, indicating that these impacts were too small to be seismically detected. The small number of craters and crater clusters that lie above the detectability line have temporal constraints that do not overlap with any known seismic events. Those impacts were deemed to have occurred during high noise periods when seismic events of that size could not be distinguished from wind noise.

### Seismology approach

**Extended Data Fig. 3:**
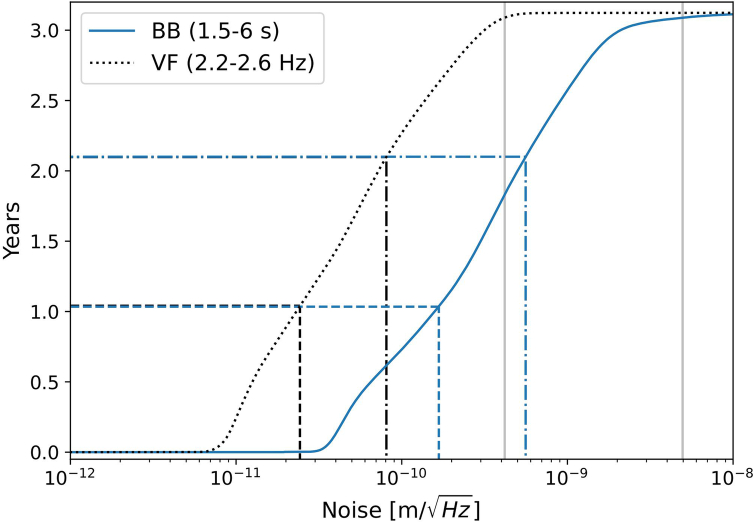
Cumulative noise distribution of the seismic data recorded by InSight until the end of June 2022 (MQS Catalog Version 12). Shown are the cumulative noise levels (in displacement per square root of Hz) in a low frequency (1.5 - 6s, blue solid line) and a high frequency (2.2 - 2.6Hz, dotted black line) band. The high frequency band is used for the VF event detection, while the low frequency band is relevant to the BB events. The dashed lines mark the 33rd noise percentile for each band (2.43 × 10^−11^m/*√*Hz VF; 1.67 × 10^−10^m/*√*Hz BB). The 67th noise percentile is given by the dash-dotted lines (8.08 × 10^−11^m/*√*Hz VF; 5.6 × 10^−10^m/*√*Hz BB), and the 99th percentile is given by the grey vertical lines (4.18 × 10^−10^m/*√*Hz VF; 4.96 × 10^−9^m/*√*Hz BB). The total recording time until end of June 2022 is 3.17 years, the 33rd noise percentile is equal to 1.03 years of low noise conditions. Noise measurements were taken every 2 minutes.

The detectability of events in the Seismology approach was based on (magnitude) equation (4), as well as event distance and magnitude considerations (Methods). Both the time and area correction used the analysis of seismic noise levels during the mission in appropriate frequency bands (Extended Data Fig. 3). We calculated the Gutenberg–Richter fit of this ‘global’ 1 yr event distribution. The resulting *a*_s_ and *b*_s_ values are converted into *k* and *b*_i_ to derive a global impact rate. This approach allows a direct conversion of a magnitude–frequency distribution into a diameter–frequency distribution. A rate per square kilometre can be obtained by dividing *k* by the area of Mars.

### Resulting impact rates

**Fig. 4:**
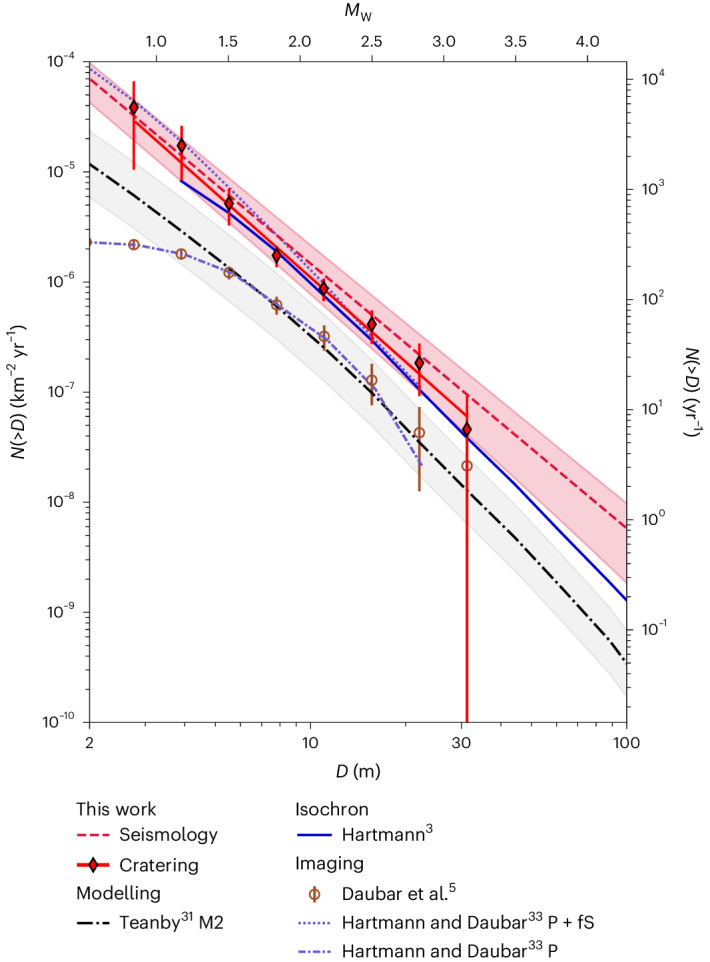
Comparison of the Martian impact rate as a cumulative SFD plot. The left and right axes show the yearly rate per square kilometre and globally, respectively. The bottom and top axes show the crater diameter and corresponding *M*_W_. Shown are results from Teanby^31^ (model 2, black dash-dotted line with grey uncertainty band), Daubar et al.^5^ (brown circles with uncertainty bars), Hartmann and Daubar^33^ (blue dotted lines, primary (P) craters; dash-dotted lines, primary and secondary (P + fS) craters) and Hartmann^3^ (dark blue solid line). The Seismology Gutenberg–Richter-derived rate is given by the red dashed line. The red uncertainty was derived by standard error propagation techniques (see Supplementary Information Section 3 for more details). The Cratering results are given by the red diamonds. Error bars were computed as sqrt(*N*) for each bin, as listed in Supplementary Table 1. The best-fitting rate is the solid red line.

The distribution of the crater diameters from the Cratering approach was binned into standard bins of width $\sqrt{2}D$ and scaled by an appropriate ATF (Methods). The cumulative crater SFD (Fig. 4) follows a power law of the form of equation (2). A least-squares fit for crater sizes 3–30 m gives *k* = 3.9 × 10^−4^ and *b*_i_ = 2.55. The Seismology approach gives *k* = 3.7 × 10^−4^ and *b*_i_ = 2.40. For a discussion on uncertainty, see Supplementary Information Section 3. A comparison of the impact rates derived here with previously published rates is given in Fig. 4, Table 1 and Supplementary Fig. 2. Our two estimates agree well with each other, within ~80 craters per year at 8 m crater diameter. The VF rate for craters *D* ≥ 8 m is, thus, ~3–4 times higher than previously estimated impact rates based on imaging (6 × 10^−7^ km^−^^2^ yr^−1^)^5^ and quite close to the prediction of ref. ^3^. (Note that the difference between these two best-fitting power laws depends on size as they have different slopes.)

**Table 1:** Comparison of the cumulative Martian impact rate between the literature and this study

Work	Method	*k* (km^−^^2^ yr^−1^)	*b*_i_	*N* ≥ 8 m (yr^−1^)	*N* ≥ 100 m (yr^−1^)
Hartmann^3^^,a^	Lunar crater isochron	7.7 × 10^−4^	2.9	276	0.19
Daubar et al.^5^^,a^	Context camera-context camera	1.2 × 10^−4^	2.5	90	—
Hartmann and Daubar^33^^,a^	Primary (imaging)	2.1 × 10^−4^	2.8	86	—
	Primary + fS (isochron)	1.1 × 10^−3^	3.0	360	—
This study	Seismology	(3.7 ± 1.7) × 10^−4^	2.40 ± 0.13	362 ± 170	0.84 ± 0.60
	Cratering	(3.9 ± 1.4) × 10^−4^	2.55 ± 0.1	280 ± 99	0.45 ± 0. 2 ^b^

^a^*k* and *b*_i_ were estimated from a least-squares fit in log-space. ^b^Extrapolated.

## Discussion and conclusions

Although only a few VF events are confirmed impacts, their similarity in seismic character and distribution in time and space suggest a common source mechanism. The VF-event rate is compatible with previous estimates of the current cratering rate, and there is no obvious indication that the VF events could easily be separated into subgroups, only one of which was impacts. Although VF events are extremely alike each other, there is a possibility that some events are not impacts. Such events could, for example, be interpreted as particularly shallow marsquakes. In the event that not all VF events are produced by impacts, our results provide an upper estimate bound to the possible current impact rate on Mars.

The slope of our cumulative impact rate is consistent between the Seismology approach at 2.40 ± 0.13 and the Cratering approach at 2.55 ± 0.1. Updated orbital imaging results suggest a slope like our results, being 2.2 for craters >8 m, although the slope varies between 1.18 and 2.55 (ref. ^28^) for different subsets. A large uncertainty in our analysis is due to the area correction, a problem known from imaging-derived rates. Specifically, it is not yet understood whether the seismic propagation model that leads to the extended coda of VF and HF events depends on a regional crustal layering that traps seismic waves. This effect makes these events stand out against others but will certainly not be sustained globally. Based on amplitude considerations, we assume this effect to end between 37° and 45° epicentral distance (see Methods for a detailed explanation).

Further, it is probable that the relationship between the crater size of an impact and the seismic moment *M*_0_ varies strongly depending on impactor material and ground strength and whether it undergoes atmospheric fragmentation. Note that our analysis provides an impact rate that may include clusters and potentially even airbursts, whereas previous rates were estimated from observed craters. Theoretically, the seismic moment and, thus, signal amplitude should be the result of a momentum transfer and, therefore, be robust against fragmentation-caused clustering, but previous studies suggest that the precise signals from crater clusters may differ notably from single impacts depending on the exact impact conditions, such as the number of fragments, separation between craters and impact trajectory^32^. For example, S1160a’s predicted diameter is slightly higher than the observed effective diameter (magenta marker on Fig. 3a), suggesting that the impact was more efficient at producing seismic waves than our scaling relationship predicts. This could indicate that the impact occurred in a more favourable target material, such as exposed bedrock. On the other hand, the predicted diameter for S0533a (another crater cluster) matches the measured effective diameter very well, possibly indicating more favourable conditions. We assume that this effect averages out in our larger dataset, and we find the agreement between our two rates obtained for 5–20 m crater sizes convincing.

Differential-imaging-based current impact rates^4,5^ have consistently been lower than the modelled rates for older surfaces^3^. Our new seismological rates are closer to the rates predicted from chronological models. Several of the fresh craters associated with InSight seismic events were found only in targeted searches, even though the area around InSight is imaged frequently. This could imply that small craters are easily missed by routine imaging than previously assumed, perhaps due to changing surface conditions, resulting in an underestimated impact rate. This holds especially for impacts that do not produce a large blast zone. The seismically derived rate is for all impacts within the radius of detectability and larger than a minimum detectable size, not a rate for the craters with associated blast zones resolved in images. Our results inform the debate on the effect of secondary craters on model ages of older surfaces^33^.

Moreover, our results do not show a roll-over at crater diameters <6 m but instead closely follow the slope of the cumulative curve of model 2 proposed by Teanby^31^, who extrapolated the orbital observations to smaller sizes based on models of atmospheric ablation and disruption of small meteoroids on Mars^30^. This suggests that the roll-over in an imaging-based crater SFD is caused by the limits of image resolution and that notable atmospheric filtering comes into effect only at even smaller impactor sizes. Seismometers, thus, probe the impact rate of smaller meteoroids than orbital observations.

The higher overall number of impacts and the higher relative number of small ones found in our study show that meteoritic impacts might be a substantial hazard for future explorations of Mars and other planets without a thick atmosphere. They also demonstrate that even a single seismic station can go a long way to quantify this risk. Due to their high corner frequency, all VF events were observed on both the larger very broadband seismometer and the smaller, more rugged, short-period sensor. This smaller sensor could be easily and affordably accommodated on any future lander anywhere in the Solar System to provide valuable measurements of the local impact rate.

## Methods

### Marsquake classification

MQS categorizes marsquakes into two main event families depending on their frequency content: low frequency and high frequency^18,19^. The events in the low-frequency family, which includes BB events, have generally been interpreted to be of tectonic origin, mostly from the Cerberus Fossae graben system, 25° to 32° epicentral distance (approximately 1,500–1,900 km) from InSight^16,17,34^. Two very distant BB events have been confirmed to be large impacts that formed craters over 100 m in diameter^14^. For most other BB events, the tectonic setting is not known, apart from one event near Valles Marineris^35^. For no other BB marsquake, including the largest one observed, S1222a, did imaging campaigns find new craters of an appropriate size^36^.

The HF family of events has been more difficult to interpret^37,38^. They are all characterized by a very extended signal duration after the seismic phases (coda) of tens of minutes, indicative of a shallow epicentre less than a few kilometres deep. This family consists of several subgroups (or types). Marsquakes in the HF-event subgroup appear to be clustered in a narrow distance range and are proposed to be related to shallow faults beneath Cerberus Fossae^17^. VF events are in the HF family.

### Background seismic noise

The background seismic noise seen at InSight is highly variable both at diurnal and seasonal scales. Following standard MQS practice^19,39^, we estimated the 33rd and 67th noise amplitude percentiles over the mission (Extended Data Fig. 3) to analyse the seismic detectability of events. These amplitudes were converted into a magnitude for each distance, using the 2.4 Hz spectral magnitude (VF magnitude equation; until 45°) and spectral magnitude (BB magnitude equation; beyond 45°) using equations from ref. ^40^:

4 $${M}_{{{{\rm{W}}}}}^{{{{\rm{VF}}}}}=\log_{10}({A}_{2.4,{{{\rm{spec}}}}})+\log_{10}(\varDelta )+11.04,$$

5 $${M}_{{{{\rm{W}}}}}^{{{{\rm{BB}}}}}=2/3\left({\rm{log}}_{10}({A}_{0})+{\rm{log}}_{10}(\varDelta )+12.6\right),$$

where *Δ* is the distance in degrees, and *A*_2.4,spec_ and *A*_0_ are the estimated percentile amplitudes for the high- and low-frequency bands, respectively. The 33rd noise percentile is 2.43 × 10^−11^ m Hz^−1/2^ for frequencies 2.2–2.6 Hz (compare 1.3 × 10^−11^ m Hz^−1/2^ for the lowest-amplitude VF). At low frequencies, 1.5–6 s, the noise amplitudes are 1.67 × 10^−10^ m Hz^−1/2^ (33rd percentile) and 5.6 × 10^−10^ m Hz^−1/2^ (67th percentile). These thresholds are marked by solid and dotted grey curves in Fig. 2a.

### Seismology event normalization

The different subsets of events used for the magnitude–frequency distribution require different correction approaches to get a complete, global set of events. The first is an area correction, the second a time correction. As the HF events have a proposed single source region, they are used as-is with respect to area (that is, we did not assume that there are other, similar source regions on Mars). For the VF events, we used a magnitude-dependent area correction. We used two different distance limits, 45° and 37°. The two furthest VF events (>37°) are more speculative, and the dataset is very sparse at this distance. The two area correction cutoff distances were taken into consideration for our uncertainty estimation. The area scaling depends on magnitude and used the solid grey curve in Fig. 2a (33rd noise percentile; equation (4) and Extended Data Fig. 3) unless the resulting distance exceeded 37°/45°, in which case, 37°/45° was used. One exception is the large VF event S0976b, which was handled in the same way as large BB events. The larger BB events *M*_W_ ≥ 3.6 (and S0976b) can be seen globally and were, thus, taken as global values. Lastly, the number of smaller BB events was multiplied by 2, as we assumed that we saw them only on the near side of Mars and that they were occurring similarly on the far side.

The time correction required an analysis of the noise. The noise recorded by SEIS is highly variable on a diurnal and seasonal scale. Most events are detectable only during periods with low wind conditions. The solid grey line in Fig. 2a shows the 33rd noise percentile, so that the noise was at or below this level 1/3 of the recorded time. Since the low magnitude VF and HF events are at or above this threshold, it is an appropriate noise level when detecting those events. Together with the 3.17 yr recording time, this gives an implicit 1/3 × 3.17 time correction for the HF and VF events when determining he rate per year (for details on the noise distribution in the relevant frequency bands, see Extended Data Fig. 3). Similarly, smaller BB events have the same time correction. However, large events of *M*_W_ ≥ 3.6 would be seen above the noise almost always. Therefore, they were divided by 3 (years) to get the yearly rate.

We calculated the Gutenberg–Richter fit to this corrected dataset for both distance cutoffs. Since the area correction itself is another source of uncertainty and influences the smaller magnitudes more strongly, we used a magnitude of completeness of *M*_W_ ≥ 2.2 and 2.5 for the VF and BBs. For the HF events, we used *M*_W_ ≥ 2.0.

### Acoustic signals in the VF coda

The VF events confirmed as impacts all contain a seismic signal called a ‘chirp’. Garcia et al.^13^ interpreted the chirp as an acoustic signal generated by the atmospheric disturbance of the meteoroid striking the ground or forming the crater. The propagation speed of the signal through the atmosphere depends on frequency. As seen in Fig. 1a (black markers), chirps arrive substantially later than other seismic phases and decrease strongly in amplitude with distance. The moveout of the speed of sound on Mars (240 m s^−1^)^41^ is consistent with the arrival times of the chirps. Evidently, acoustic signals arrive much later for more distant events and only close events are expected to have chirps associated within the event coda. No other event of any type has a clear associated chirp except for the confirmed impact VF events.

In Fig. 2a, events with and without chirps can be separated by a line, indicating that the presence of chirps depends on distance and magnitude. This strongly suggests that the presence of a chirp in the VF signal is not a prerequisite for the signal to be of impact origin. Indeed, neither of the two large impacts detected at teleseismic distances produced a signal with a discernible acoustic arrival^14^. Supplementary Figs. 3 and 4 show spectrograms of VF events without and with chirps, respectively.

### Spectral character of VF events

We estimated whether the spectra of VF events are notably different from other event types in terms of their corner frequency. The corner frequency *f*_c_ describes where the displacement spectrum of a seismic signal deviates from a flat plateau and is inversely related to the duration of the source process. For tectonic events on Earth, a general tendency of ${f}_\mathrm{c}\propto {M}_{0}^{-3}$ is observed^42^, with a scatter of about an order of magnitude in *f*_c_ due to variations in stress drop and fault geometry. On Mars, a ${f}_\mathrm{c}\propto {M}_{0}^{-3}$ relation is observed between low-frequency, BB and HF events, with exceptions for many low-frequency and BB marsquakes in Cerberus Fossae^17^. The VF events deviate from this relation and form their own cluster of events (see triangles in Extended Data Fig. 2). This cluster has a corner frequency. That is, the event duration is much higher than for tectonic events of the same magnitude, compatible with a hypervelocity impact^14^.

### Event magnitudes and spectral fits for S1034a and S1160a

The magnitudes of VF events were predominantly calculated from the 2.4 Hz spectral peak (equation (4))^40^. A few VF events have a long period energy above the noise level, and the magnitude can, thus, be calculated from the flat part of the event spectrum. One event, S1034a, has an unusually large signal amplification at the 2.4 Hz resonance peak. The spectral fit puts it over 50 dB above the noise window (on a rather windy Martian day). Other large VF events have amplifications of 30 dB or lower over the noise (for example, S0976b and S0794a). This unusual amplification is also not reflected in the flat part of the spectrum and is probably an effect of the very close proximity of the event and interactions with the amplifying subsurface structure. We used a fit of the flat part of the spectrum to get *A*_0_ and used the appropriate equation from Böse et al.^40^:

6 $${M}_{{{{\rm{W}}}},{{{\rm{spec}}}},{{{\rm{HF}}}}}=2/3\left({\rm{log}}_{10}({A}_{0})+0.8{\rm{log}}_{10}(\varDelta )+12.8\right),$$

which changes the magnitude from 3.0 (MQS catalogue) to 2.1.

The catalogue magnitude of event S1160a has been calculated from the chirp (×1 pick) and not from Pg or Sg. Using a spectral window around the Pg and Sg arrivals, we obtained a magnitude of 1.3 (down from 1.5). Supplementary Fig. 5 shows the spectral fit for both events.

### Spatial and temporal distributions of VF events

For no single VF or HF event could a back azimuth, that is the direction as seen from InSight, be determined from the P and S waves alone. So, only the distance distribution could be analysed. We found a strong difference between the distributions for HF and VF distance (Fig. 2a). HF events are mostly clustered between 20° and 30° (~1,180–1,775 km), consistent with a common location in the region of the seismically active Cerberus Fossae graben system^17^. VF events appear to be unclustered with respect to distance and have been detected as far away as 45° (~2,600 km). Within their detection threshold, the spatial distribution of VF events of a given size is distinctly more uniform over the Martian surface than for other event classes. In particular, some VF events seem to originate very close to the InSight lander (less than 1° separation for event S1034a), much closer than other event types. Very weak VF events could be misclassified as an HF event if the VF energy is difficult to differentiate from wind, as apparent by the gap in VF events at the HF cluster in Fig. 2.

Additionally, we determined whether the temporal variability of VF events is also consistent with the expected variation in impact rate over time. Although a seasonal variation in impact rate has been hypothesized^9,43^, the magnitude of that variability is uncertain and could be within the error bars of the observations. More VF events were detected in Martian year 2 of the InSight mission than year 1 (as have other event types). However, taking the seasonal variability of the background noise into account, the differences in detection counts between year 1 and year 2 are within one standard deviation of what would be expected for an event rate that is constant during the whole time as a Poisson process (Supplementary Information Section 1). This is in contrast to HF events, which have a strong seasonal variability^39^.

### Impact momentum scaling

A set of seismically detected impacts on several planetary bodies shows that the peak P-wave amplitude (${v}_{\max }$ in m s^−1^) scaled by impactor momentum (*p*_i_ in N s) produces a clear trend with distance from source, where the scaled velocity drops as *x*^−1.56^ (where *x* in kilometres is scaled to 1 km). As demonstrated in Garcia et al.^13^, the resulting scaling relationship is

7 $${v}_{\max }\left(\frac{1{0}^{6}\,{{\mbox{N}\,\mathrm{s}}}\,}{{p}_{{{{\rm{i}}}}}}\right)=1.1\times 1{0}^{-5}{\left(\frac{x}{1\,{{\mbox{km}}}}\right)}^{-1.56}.$$

Numerical modelling studies have shown that the seismic moment *M*_0_ scales linearly with the impact momentum^26,27^ and as a power law with crater diameter *M*_0_ = *cD*^*n*^, where *n* varies between 3.0 and 3.6 depending on target material properties. For this work, we chose *n* = 3.3 to represent an average between strongly cohesive material and a cohesionless sand. As *M*_0_ ∝ *D*^*n*^ (equation (3), where *n* = 3.3), it follows that *v* ∝ *D*^*n*^*x*^−1.56^. The impact detectability curve can, therefore, be defined as the maximum distance (${x}_{\max }$) at which a crater of diameter *D* would be detectable by InSight:

8 $${x}_{\max }=a{D}^{n/1.56},$$

where *a* is the vertical offset in the log–log plot, determined by setting the curve to match the detectable diameter defined by the detection curve for VF events (equation (4)) at a distance of 1,000 km, which returns a crater diameter of 6.4 m. The absolute value of the peak P-wave amplitude is contained in the constant *a*, which eliminates the need to explicitly relate ${v}_{\max }$ to *A*_2.4,spec_. The resulting detectability curve is therefore

9 $${x}_{\max }=15.4{D}^{n/1.56}.$$

### Cratering event selection

In the Cratering method, we considered only VF events because they produce craters in the size range of the numerical simulations that underpin the relationship in equation (3). Impacts that produce larger craters, such as those associated with BB events, will release their seismic moment at a substantially greater depth than most VF-event impacts. In this case, the seismic moment used in equation (3) should be corrected to account for the difference in density and the wave speed as a function of release^14^. As equation (3) underestimates crater sizes for BB events, we excluded all BB events and the largest VF event from the following calculations.

The self-consistency of this approach is confirmed by the fact that crater sizes for five out of six nearby impacts identified in InSight data were predicted correctly to within uncertainty, as shown in Fig. 3.

### Impact rate normalization

The distribution of crater diameters derived from the VF events using the Cratering approach was binned into bins of width $\sqrt{2}D$ (after ref. ^3^). This resulted in incremental counts, as shown in Supplementary Table 1. To convert these counts into impact rates, we scaled them by an appropriate ATF that reflects the relevant area and time of detection^5^. The detection area for each diameter bin was, therefore, calculated as the spherical segment area around InSight in which a crater with the central diameter of a given bin would be detectable, using equation (9), up to a distance of 45° (~2,600 km). This resulted in an overall impact rate per square kilometre. The detection time factor was determined by the temporal variation of the seismic noise. The seismic data recorded by InSight were highly affected by the daily and seasonal wind patterns^44,45^. Small to intermediate-sized events can be detected 1/3 of the recording time (Fig. 2 and Extended Data Fig. 3), amounting to a total of 378 d. Hence, to derive the final incremental impact rate per year per square kilometre, we divided the rate per unit area by 1.03 yr. The final ATF for each bin is given in Supplementary Table 1, along with crater counts for each bin, as computed from the dataset of VF events. Finally, the incremental impact rate per year per square kilometre was summed cumulatively to produce the cumulative impact rate per year per square kilometre.

## Supplementary information

Supplementary Information Supplementary Figs. 1–7, Table 1 and discussion.

## Figures and Tables

**Extended Data Table 1:**
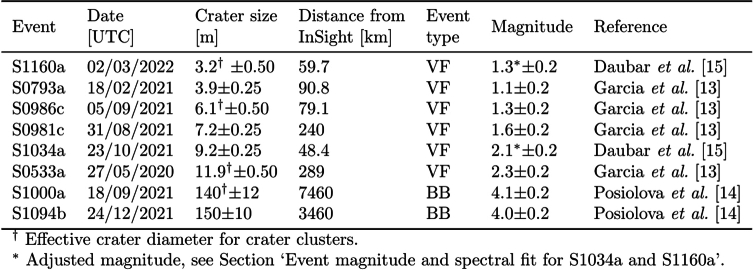
Table listing seismic events and corresponding impact crater information sorted by distance to the InSight landing site^13–15^

The uncertainties in crater sizes are given by the resolution of HiRISE images. Magnitudes are calculated using the corresponding crater distance and can differ from the MQS Catalog^21^.

## Data Availability

The InSight seismic event catalogue v.12 is at 10.12686/a18. InSight SEIS data^46^ are referenced at 10.18715/SEIS.INSIGHT.XB_2016 and are available from the IRIS Data Management Center, Paris Institute of Earth Physics Data Center and the NASA Planetary Data System.
