# Supplementary material for: An estimate of the impact rate on Mars from statistics of very-high-frequency marsquakes
Source: Nat Astron. 2024 Jun 28;8(9):1138–47. doi: 10.1038/s41550-024-02301-z (PMC11410648; doi:10.1038/s41550-024-02301-z)
Supplement: Supplementary file 1 — Supplementary Figs. 1–7, Table 1 and discussion. [file 41550_2024_2301_MOESM1_ESM.pdf]

nature astronomy

Supplementary information https://doi.org/10.1038/s41550-024-02301-z

An estimate of the impact rate on Mars from statistics of very-high-frequency marsquakes

In the format provided by the authors and unedited

Supplementary Material for
A new estimate of the impact rate on Mars from Very High Frequency marsquake statistics

Géraldine Zenhäusern, Natalia Wójcicka, Simon C. Stähler, Gareth S. Collins, Ingrid J. Daubar, Martin Knapmeyer, Savas Ceylan, John F. Clinton, Domenico Giardini

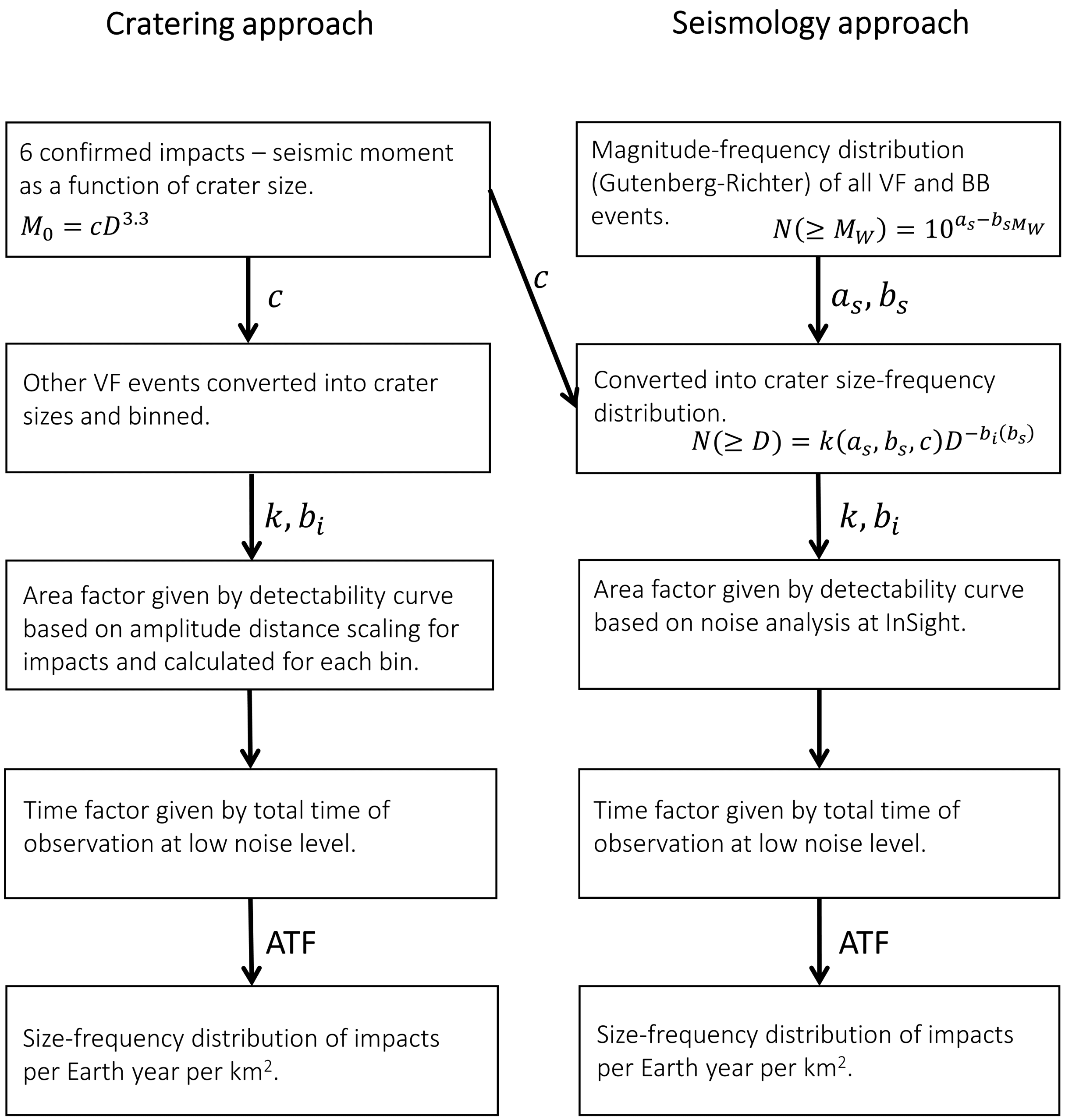

Supplementary Material Figure 1: A flowchart illustrating the two methods presented in this study.

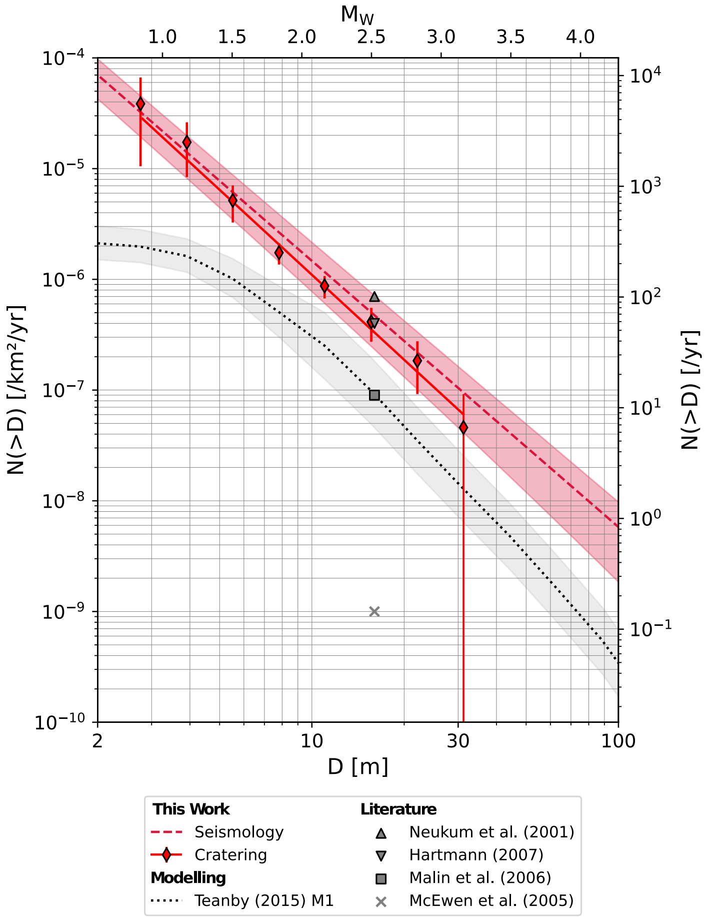

Supplementary Material Figure 2: Martian impact rate comparison as a cumulative size-frequency distribution (SFD) plot. The left and right axes show the yearly rate per km^2 and globally, respectively. The bottom and top axes show the crater diameter and corresponding M_{W}. Shown are values N($\geq$ 16 m) for Neukum *et al.* [49] (grey upwards triangle), Hartmann [50] (grey downwards triangle), Malin *et al.* [4] (grey square), and McEwen *et al.* [51] (grey cross), as given by Hartmann [50]. Additionally shown is Teanby [30] (model 1, dotted line with grey uncertainty band). The 'Seismology' Gutenberg-Richter-derived rate is given by the red dashed line with red uncertainty derived by standard error propagation techniques (see Supplementary Section 3 for more details). The 'Cratering' results are given by the red diamonds with errorbars (computed as $sqrt(N)$ for each bin, as listed in Supplementary Table 1), with best-fit rate (solid red line).

1 Occurrence rate of VF events

Since the wind noise recorded by InSight is prohibitive to almost all event detections during daytime in spring and summer, and to detections at all times of day during winter, a comparison of event rates between different times of Martian year, or between several years, requires to take the respective noise amplitudes into account: The catalogued events reflect only the *detection* rate of events, but not their *occurrence* rate. We intend to answer if the data supports that the VF events *occur* at a constant rate, although our *detection* rate appears to vary strongly over time. Since any single realization of a stochastic process shows fluctuations compared to the expectation, we need to answer if the observed deviation from a stationary Poisson process is statistically significant, i.e. if it is so unlikely that it contradicts the assumption of a constant occurrence rate.

A methodology to assess the effect of the variable noise background was developed by Knapmeyer *et al.*

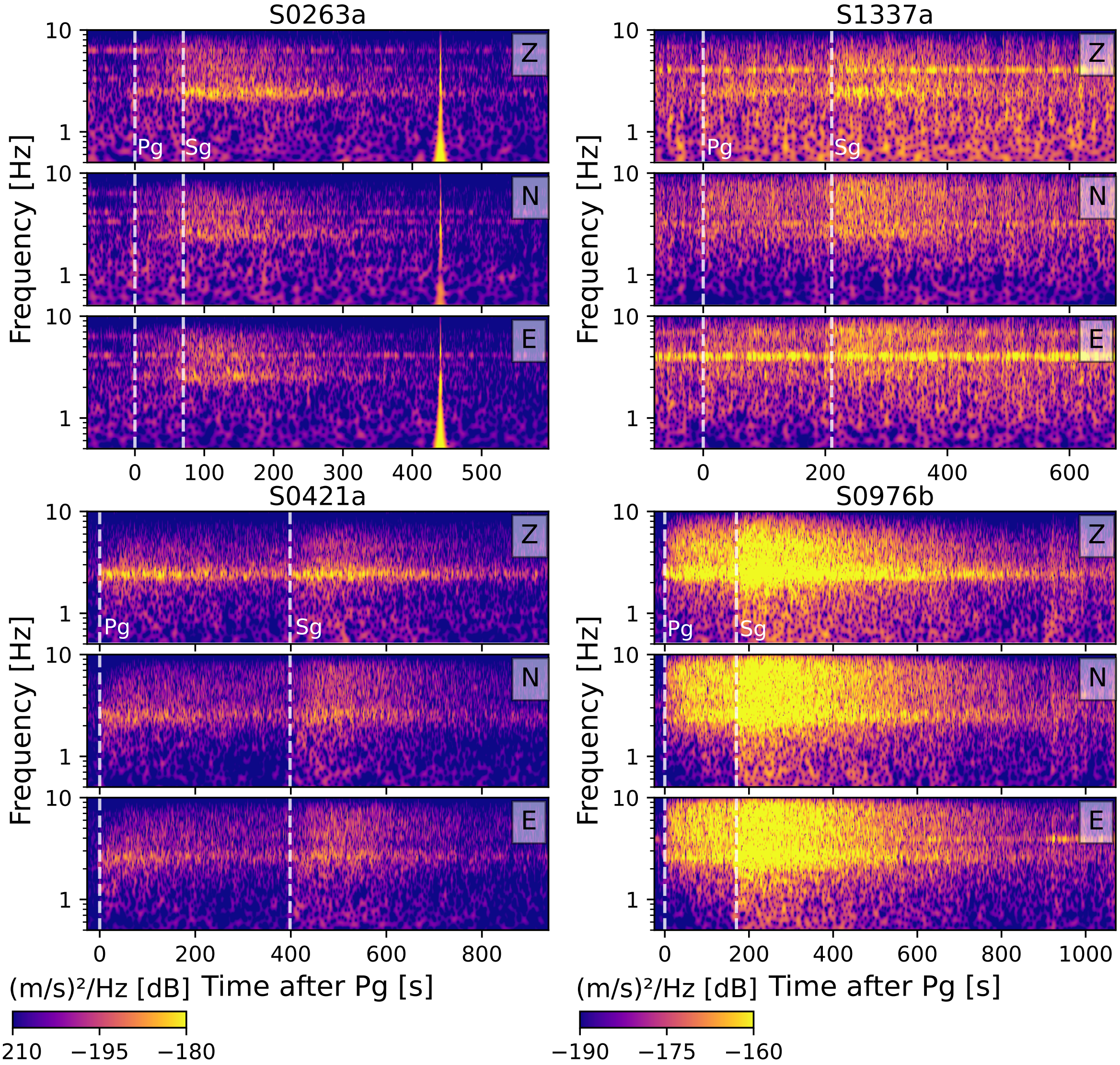

Supplementary Material Figure 3: Velocity spectrograms for events S0263a, S0421a, S0976b, and S1337a. Phase picks are marked by vertical dashed lines. These events have epicentral distances >5 ° and are not currently associated with an impact crater. Note the different amplitudes for the left and right column.

[39] to demonstrate the seasonal variability of the occurrence rate of HF events. If the recorded amplitudes of events follow a power law with a slope b, and if the catalog is complete for events with recorded amplitudes exceeding a certain threshold amplitude A_{TH}, then one can predict the number of events that would be missed if the background noise level increases: The signal-to-noise (SNR) ratio required for a certain detection dictates that A_{TH} increases by the same factor as the noise level, and the number of events lost due to higher noise then depends on b. A detailed mathematical description of how to compute a short-term detection efficiency from noise RMS amplitudes, and how to average short time detection efficiencies to obtain daily or long-term average detection efficiencies, is given in section S4 of the supplement of Knapmeyer *et al.* [39]. Said supplement also shows an application where the detection rate of HF events during the noisier second half of the night is predicted from the detections during the less noisy first half (and vice versa).

In the present work, we consider the 58 VF events of qualities A, B, and C that were detected up to

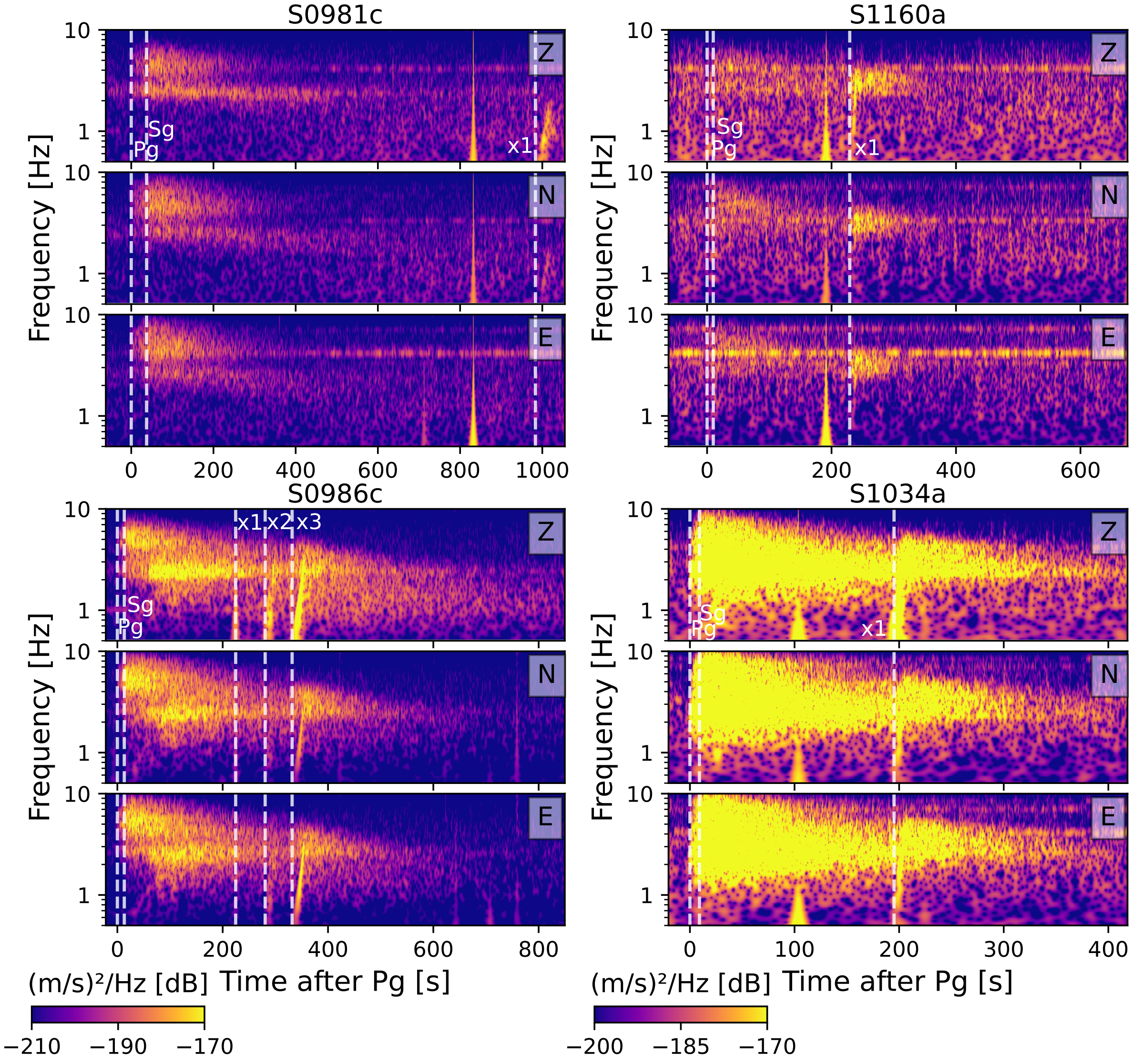

Supplementary Material Figure 4: Velocity spectrograms for events S0981c, S0986c, S01034a, and S1160a. Phase picks are marked by vertical dashed lines. All four events have been identified as impacts [13, 15]. Note the different amplitudes for the left and right column.

May 11th 2022, or Sol 1228. The distributions of recorded amplitudes and SNR suggest that the catalog is representative for all events with amplitudes higher than -200 dB (displacement with respect to 1 m) and with SNR exceeding 2.9. Of the recorded VF events, 34 are thus representative for the actual VF event population (Since we know that the catalog is not complete due to the high noises at some times, it can only be representative). The cumulative number of detected events is shown as squares in Supplementary Material Figure 6a.

When testing if the data makes a certain occurrence process of events plausible, we have to keep in mind that the *actual* event process, together with the observed noise conditions, resulted in the actual cumulative number of detections. Whatever we assume about the production of events must reproduce the observation at least during the time interval from which we derive the process parameters.

We will assume that events *occur* according to a stationary Poisson process. We will also assume that the

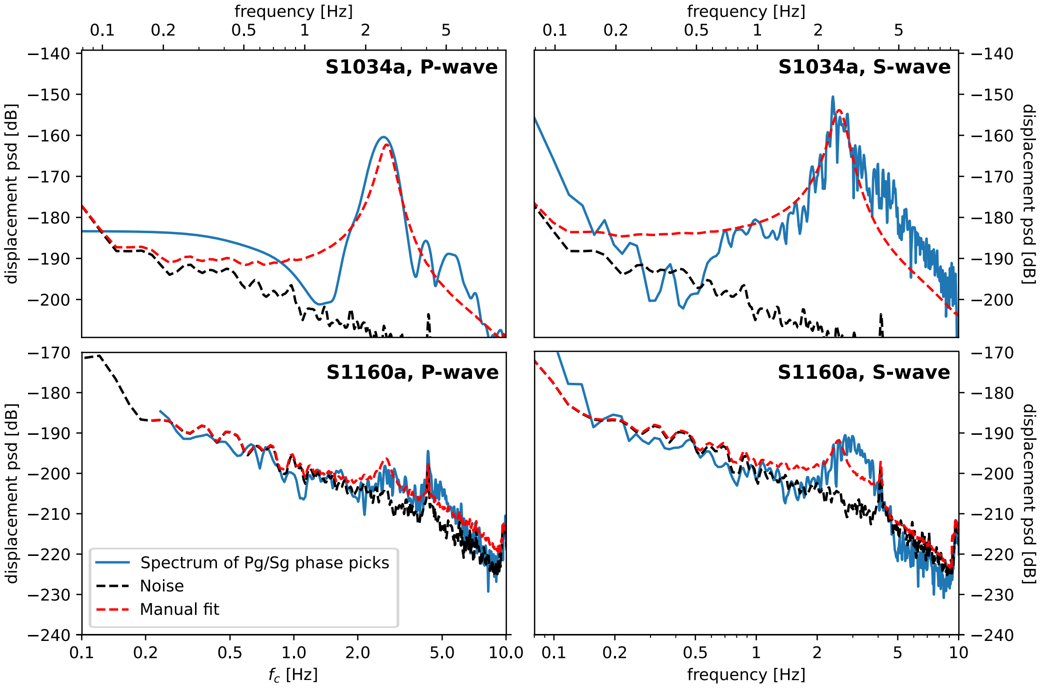

Supplementary Material Figure 5: Spectral fits used to determine magnitudes for nearby meteoritic impacts S1034a and S1160a. The black dashed line marks the ambient noise before the event and is used to estimate the frequency window in which a fit can be done. The blue line marks the spectra in a 20 s window around the phase pick, manually selected to exclude wind or other obvious artefacts. The red line is a best fit of a Brune function, superposed with a Lorenz term for the local 2.4 Hz resonance [33] added to the pre-event noise. The long period flat part of this fit function minus noise is used to determine the event magnitude as described in Böse *et al.* [40]. Note that the 'S-wave' window for S1160a is on the chirp of the event.

Supplementary Material Table 1: Binned incremental and cumulative crater counts computed from VF events, and the respective Area Time Factor (ATF) used for each bin. The final column gives the scaled cumulative values plotted in Fig. 4.

Bin minimum $D_{\min}$ [m]	Bin maximum $D_{\max}$ [m]	$N(D_{\min} \leq D < D_{\max})$	$N(\geq D)$	ATF [km^{2}yr]	$N(\geq D)$ [km^{-2}yr^{-1}]
2.76	3.90	4	58	1.89×10^5	3.85×10^{-5}
3.90	5.53	10	54	8.24×10^6	1.73×10^{-5}
5.53	7.80	12	45	3.15×10^6	5.16×10^{-6}
7.80	11.05	13	31	1.49×10^7	1.74×10^{-6}
11.05	15.56	10	19	2.18×10^7	8.72×10^{-7}
15.56	22.08	5	7	2.18×10^7	4.13×10^{-7}
22.08	31.23	3	3	2.18×10^7	1.84×10^{-7}
31.23	44.17	1	1	2.18×10^7	4.59×10^{-8}

only time dependent modification of detection probability is solely due to the background noise and not, for example, due to changes of the personnel in charge (the InSight team conducts a multi-stage review process to account for all kinds of operative changes). A statistically significant deviation between observed and predicted detections will point to either an incorrect rate estimation, or a non-stationary process. Absence or insignificance will not prove that our assumption is correct, but also not give a reason to doubt it.

The procedure of our test is as follows:

1. Estimate the *detection* rate of events assuming a stationary Poisson process. The maximum likelihood estimation for the reference time window (RTW, 28 events from sol 288 to sol 1100) results in a detection rate of 0.03448 events per sol. The RTW covers the time between two major data gaps to a priori exclude possibly lost detections during the gaps.

2. Plot the observed cumulative number of detections (squares in Supplementary Material Fig. 6a) and the cumulative number expected from the rate estimate (dashed blue line in Supplementary Material Fig. 6a extrapolates beyond the limits of the RTW)

3. Compute the *daily* detection efficiency for the entire time as described by Knapmeyer *et al.* [39]. The result is shown as solid red curve in Supplementary Material Figure 6a. Note the logarithmic scale.

4. Average the detection efficiency over the entire RTW. The resulting long-term average detection efficiency is 0.83227.

5. Estimate the *occurrence* rate of events assuming that the occurrence follows a stationary Poisson process: 0.03448/0.83227=0.04143 events per sol, suggesting that 33 to 34 events actually occurred during the RTW. In the following, we assume that events occur according to a stationary Poisson process with this event rate.

6. Compute the expected cumulative number of detections from the stationary occurrence rate (0.04143) and the daily average detection efficiency (solid blue line in Supplementary Material Fig. 6a).

7. Plot as symbols in 6b the difference ("residual") between the observed cumulative number of detections (squares in Supplementary Material Fig. 6a) and the cumulative number predicted from stationary occurrence rate and daily detection efficiency (solid blue line in Supplementary Material Fig. 6a).

8. Numerically estimate by how much the predicted detection rate, estimated from the assumed stationary occurrence rate and accounting for the daily detection efficiency, scatters, i.e. estimate the distribution of the cumulative detection count at any time. To this end, generate a random sequence with the estimated occurence rate, and modulate it according to the time-dependent detection efficiency. From the *synthetic* cumulative detection count compute an apparent stationary rate, and the deviation of the synthetic cumulative detection count from the expectation resulting from the apparent rate. The gray lines in Supplementary Material Fig. 6b indicate the deviation from the expected value in terms of quantiles for commonly used probabilities (e.g. the 68.28 quantile indicates that 68.28% of the realizations result in values between the respective lines. Probabilities correspond to 1,2,3, and 4 standard deviations of a gaussian distribution, i.e. 0.6827, 0.9545, 0.9973, and 0.9999, respectively).

9. Compare the deviation of the observed cumulative event count from a stationary Poisson process to the distribution obtained in step 8. To this end, we extract the probability to observe a deviation like the observed for the times of all events and plot it as function of time (Supplementary Material Fig. 7). We do not derive a single number as significance criterion but draw a conservative conclusion from visual inspection of the resulting graph.

We find that the deviation of the cumulative event count from the expectation for a stationary Poisson process reaches statistically significant amounts during the first martian year (sols 288 to 687) of InSight's operation when calibrating the stationary process using events from Sol 288 to Sol 1100. The actual probability of this deviation depends on the reference time window chosen, as the distribution becomes wide at long times before or after the RTW. Tests with alternative RTWs from Sol 288 to Sol 500, and from Sol 800 to Sol 1100, however suggest that the significance of the deviation is not an artefact of the window from sol 288 to 1100. A change of detection rate was also observed for the class of HF events [39] and, to a lesser extent, LF events [16, 21], where the event rate apparently increased. The reason for this is unknown, but since the study of Dahmen *et al.* [21] is based on a machine learning technique, a change of MQS practices or personnel can be excluded as explanation of the detection rate increase.

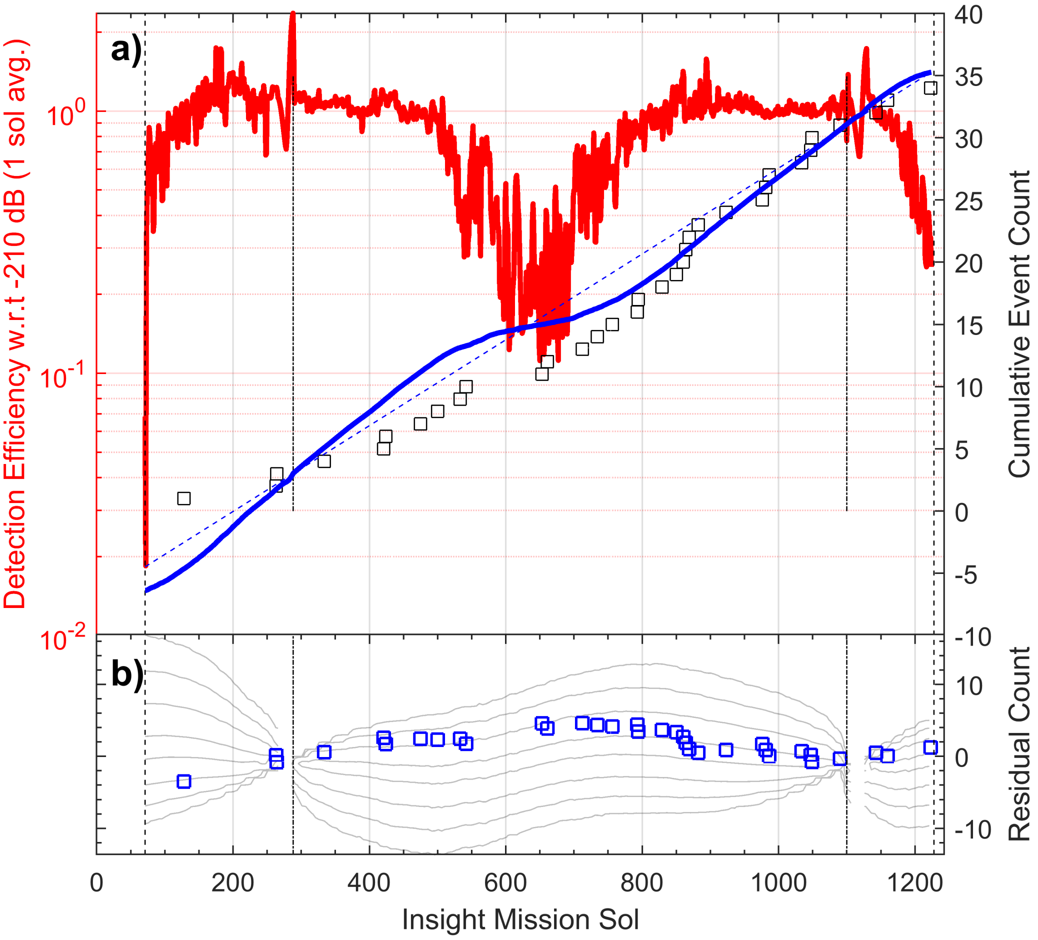

Supplementary Material Figure 6: Effect of background noise on VF event rate. (a) Cumulative event count and expected modification of a stationary Poisson process due to noise. Squares: cumulative count of detected events. Blue dashed line: expected count for a stationary Poisson process with rate derived from time window marked by vertical dashed lines. Red solid line: Per-sol averaged detection efficiency due to background noise. Blue solid line: Modification of the cumulative count expected from a stationary Poisson process due to variable detection efficiency. (b) Residual count w.r.t constant occurrence rate. Squares: Difference between observed cumulative count (squares in (a)) and cumulative count predicted from efficiency-modulated stationary Poisson process (occurrence rate from blue dashed line in (a), divided by average detection efficiency). Grey lines and labels: Probability that the variability between realizations of the modulated stationary Poisson process results in a difference between the corresponding lines. Contours result from a Monte Carlo experiment with ten million synthetic event sequences and represent probabilities of 68.3%, 95.5%, 99.7%, and 99.99%, respectively. Note that these lines come in pairs, the outermost lines correspond to 99.99%.

2 Conversion of impacts power law and Gutenberg-Richter law

Recalling the Gutenberg-Richter law [Eq. 1; e.g. 22]

$$N(\geq M_{\mathrm{W}}) = 10^{a_{\mathrm{s}} - b_{\mathrm{s}} M_{\mathrm{W}}}, \tag{2.1}$$

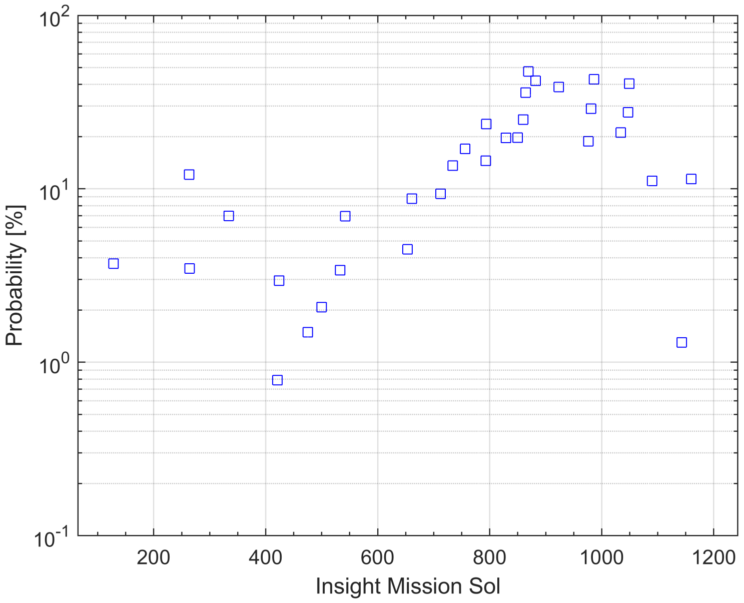

Supplementary Material Figure 7: Probability to observe a cumulative count as high as the actual observation at the times of the observations, obtained by reading probabilities p to not exceed a certain residual from Figure 6b and then plotting 1-p. Note that the observed residuals have a probability of less than 5% during much of the first martian year (i.e. up to Sol 687).

where M_W is the moment magnitude, N the number of events, and a_s and b_s describe the productivity and slope of the distribution, respectively. Similarly recalling the impacts power law [Eq. 2; e.g. 49]

$$N(\geq D) = kD^{-b_i}, \tag{2.2}$$

where N is the number of impacts, D is the diameter, k the productivity, and b_i the slope.

Taking the logarithm of Eq. 2.1 and using M_0 instead of M_W gives

$$\log_{10} N(\geq M_0) = a_s - \frac{2}{3} b_s \left(\log_{10} M_0 - 9.1\right). \tag{2.3}$$

Inserting the M_0 - diameter relationship (Eq. 3) and re-ordering the equation produces

$$\log_{10} N(\geq D) = a_s + \frac{2}{3} 9.1 b_s - \frac{2}{3} b_s \log_{10}\left(cD^{3.3}\right). \tag{2.4}$$

Splitting the last term gives

$$\log_{10} N(\geq D) = a_s - \frac{2}{3} b_s \left(\log_{10} c - 9.1\right) - 3.3\frac{2}{3} b_s \log_{10} D. \tag{2.5}$$

Taking the logarithm of Eq. 2.2 gives

$$\log_{10} N(\geq D) = \log_{10} k - b_i \log_{10} D. \tag{2.6}$$

Comparing the form of Eq. 2.6 to Eq. 2.5 results in

$$\begin{aligned} \log_{10} k &= a_s - \frac{2}{3} b_s \left(\log_{10} c - 9.1\right) \\ b_i &= 3.3\frac{2}{3} b_s. \end{aligned} \tag{2.7}$$

Finally, the impacts power law coefficients can be expressed as

$$\begin{aligned} k &= 10^{a_s - \frac{2}{3} b_\mathrm{s} (\log_{10} c - 9.1)} \\ b_\mathrm{i} &= 2.2 b_\mathrm{s}. \end{aligned} \tag{2.8}$$

Conversely, the Gutenberg-Richter coefficients can be expressed as

$$\begin{aligned} a_\mathrm{s} &= \log_{10} k + \frac{b_\mathrm{i}}{3.3} (\log_{10} c - 9.1) \\ b_\mathrm{s} &= \frac{b_\mathrm{i}}{2.2}. \end{aligned} \tag{2.9}$$

3 Rate uncertainty

The uncertainty of the impact rates is different between the two methods deployed in this study.

For 'Seismology', three different parameters contribute to the final uncertainty: a_s, b_s, and c. a_s and b_s are derived from the four Gutenberg-Richter fits visible in Fig. 2b by calculating the mean and standard deviation for each parameter. These fits are obtained using different magnitudes of completeness and maximum distances for the area correction. a_s is obtained from anchoring the fit to the event rate at the respective magnitude of completeness. This gives $a_s = 4.59 \pm 0.16$ and $b_s = 1.09 \pm 0.06$. Finally, $c = (8.1 \pm 3.0) \times 10^8$ (cf. Eq. 3, Fig. 3). Inserting expressions 2.8 into the impacts SFD $N(\geq D) = kD^{-b_i}$ allows the derivation of the partial derivative of N with respect to a_s, b_s, and c. This can then be used to calculate the uncertainty of the form

$$\sigma_N = \sqrt{\left(\frac{\partial N}{\partial a_s}\sigma_{a_s}\right)^2 + \left(\frac{\partial N}{\partial b_s}\sigma_{b_s}\right)^2 + \left(\frac{\partial N}{\partial c}\sigma_c\right)^2}, \tag{3.1}$$

where σ are the uncertainty for the rate N, and parameters a_s, b_s, and c. Since a_s and b_s cannot be considered independent, this represents an indicative lower bound estimate.

For the 'Cratering' method, the uncertainty in the crater size predicted for each event is determined by the uncertainties in seismic moment (σ_M) and the proportionality constant c (σ_c). These uncertainties are combined using standard error propagation methods.

The error bars in crater counts in each bin shown in Fig. 4 are given by $\sqrt{N}$, which is a standard procedure for binned data. Eq. 2 is then fitted to the binned data using least squares algorithm in log space. This method produces uncertainties in both k and b_i. These errors can be combined using standard error propagation to calculate the total uncertainty in the number of craters larger than 8 m across all of Mars, as follows:

$$\sigma_{N(>8\mathrm{m})} = \sqrt{\left(\frac{\partial N}{\partial k}\sigma_k\right)^2 + \left(\frac{\partial N}{\partial b_i}\sigma_{b_i}\right)^2 + \left(\frac{\partial N}{\partial c}\sigma_c\right)^2}. \tag{3.2}$$

Because the value of N does not directly depend on the value of c, it is included in these calculations by rewriting Eq. 2 in terms of M and evaluating the partial derivative of N w.r.t. c. This results in uncertainty in $N(\geq 8)$ quoted in Table 1 of ± 99 craters. Results from both methods still agree with each other within the error margin.

References:

4. Malin, M. C., Edgett, K. S., Posiolova, L. V., McColley, S. M. & Dobrea, E. Z. N. Present-Day Impact Cratering Rate and Contemporary Gully Activity on Mars. *Science* **314,** 1573–1577. doi:10.1126/science.1135156 (2006).
13. Garcia, R. *et al.* Newly formed craters on Mars located using seismic and acoustic wave data from InSight. *Nat. Geosci.* **15,** 774–780. doi:10.1038/s41561-022-01014-0 (Oct. 2022).
15. Daubar, I. J. *et al.* Two Seismic Events from InSight Confirmed as New Impacts on Mars. *Planet. Sci. J.* **4,** 175. doi:10.3847/PSJ/ace9b4 (Sept. 2023).

16. Ceylan, S. *et al.* The marsquake catalogue from InSight, sols 0–1011. *Phys. Earth Planet. Inter.,* 106943. doi:10.1016/j.pepi.2022.106943 (2022).

21. Dahmen, N. L. *et al.* MarsQuakeNet: A More Complete Marsquake Catalog Obtained by Deep Learning Techniques. *J. Geophys. Res. Planets* **127,** e2022JE007503. doi:10.1029/2022JE007503 (Nov. 2022).

22. Gutenberg, B. & Richter, C. F. Frequency of earthquakes in California. *Bull. Seismol. Soc. Am.* **34,** 185–188. doi:10.1785/BSSA0340040185 (1944).

30. Teanby, N. A. Predicted detection rates of regional-scale meteorite impacts on Mars with the InSight short-period seismometer. *Icarus* **256,** 49–62. doi:10.1016/j.icarus.2015.04.012 (Aug. 2015).

33. Giardini, D. *et al.* The seismicity of Mars. *Nat. Geosci.* **13,** 205–212. doi:10.1038/s41561-020-0539-8 (Mar. 2020).

39. Knapmeyer, M. *et al.* Seasonal seismic activity on Mars. *Earth Planet. Sci. Lett.* **576,** 117171. doi:10.1016/j.epsl.2021.117171 (2021).

40. Böse, M. *et al.* Magnitude Scales for Marsquakes Calibrated from InSight Data. *Bull. Seismol. Soc. Am.* **111,** 3003–3015. doi:10.1785/0120210045 (June 2021).

49. Neukum, G., Ivanov, B. A. & Hartmann, W. K. Cratering Records in the Inner Solar System in Relation to the Lunar Reference System. *Space Sci. Rev.* **96,** 55–86. doi:10.1023/A:1011989004263 (Apr. 2001).

50. Hartmann, W. K. Martian cratering 9: Toward resolution of the controversy about small craters. *Icarus* **189,** 274–278. doi:10.1016/j.icarus.2007.02.011 (2007).

51. McEwen, A. S. *et al.* The rayed crater Zunil and interpretations of small impact craters on Mars. *Icarus* **176,** 351–381. doi:10.1016/j.icarus.2005.02.009 (2005).
